# Geniculate Artery Embolization for Recurrent Effusion After Total Knee Arthroplasty in a Patient With Hemophilia

**DOI:** 10.7759/cureus.109289

**Published:** 2026-05-20

**Authors:** Miranda Czymek, Rex W Lutz, Christopher Kim, Zachary D Post, Alvin Ong

**Affiliations:** 1 Orthopedics, Rowan-Virtua School of Osteopathic Medicine, Stratford, USA; 2 Orthopedic Surgery, Jefferson Health New Jersey, Stratford, USA; 3 Interventional Radiology, Atlantic Medical Imaging, Galloway, USA; 4 Orthopedic Surgery, Rothman Orthopaedic Institute, Egg Harbor Township, USA

**Keywords:** hemophelia, ir guided embolization, knee arthroplasty, post-op, recurrent effusion

## Abstract

Treatment options are limited in those with end-stage hemophilic arthropathy. Total knee arthroplasty (TKA) serves as a therapeutic and definitive treatment for hemophilic arthropathy, yet patients with hemophilia (PWH) are at increased risk of recurrent hemarthrosis following TKA. Standardized treatment of recurrent effusion (RE) after TKA has not been well-established.

In this case presentation, a 62-year-old male with hemophilia A presented with RE after TKA for end-stage hemophilic arthropathy. After undergoing conservative management and an infectious workup, the patient was referred to interventional radiology for geniculate artery embolization (GAE). Four arteries near hemostasis were achieved. The patient's outcomes were evaluated using the Western Ontario and McMaster Osteoarthritis Index (WOMAC). At the three-month follow-up from embolization, the patient had an 83% decrease in the WOMAC score and no recurrence of effusion.

## Introduction

Hemophilia A is a genetic disorder caused by a deficiency in coagulation factor VIII. The severity of symptoms correlates with the level of coagulation factor VIII produced by the affected individual, which includes prolonged bleeding times and bleeding in atypical locations such as the joint space. Hemarthrosis is the most common bleeding location, reported in up to 80% of all hemophiliac hemorrhages [1]. Patients with hemophilia who undergo total hip or knee arthroplasty (TKA) have a higher rate of unplanned 30-day readmission due to bleeding [2]. A meta-analysis conducted by Fenelon et al. found the most common complication following TKA in patients with hemophilia (PWH) was hematoma/hemarthrosis at a rate of 7.6%, compared to non-hemophilia patients whose rates are roughly 0.3% to 1.6% [3]. Recurrent effusion (RE) as hemarthrosis leads to stiffness, decreased function, and, in some cases, infection [4-6]. Standard treatment of RE has not been established; however, growing evidence shows geniculate artery embolization (GAE) as a viable option for RE after TKA [7]. GAE is an interventional radiology technique in which selective geniculate arteries are embolized to reduce RE. In this case, we demonstrate the complete resolution of RE and improved patient-reported outcomes utilizing GAE in a PWH.

## Case presentation

A 62-year-old male with a past medical history of aortic stenosis, seizure disorder, depression, bipolar disorder, hypertension, and hemophilia A treated with emicizumab presented to the orthopedic clinic status post TKA in 2016 for post-traumatic arthritis. He was status post open reduction and internal fixation of a distal femur fracture with a lateral locking plate following a motor vehicle accident in 2011. The patient complained of RE every three to four months following TKA, leading to swelling, pain, and decreased function. The patient denied any history of recent trauma or constitutional symptoms indicating infection. 

**Figure 1:**
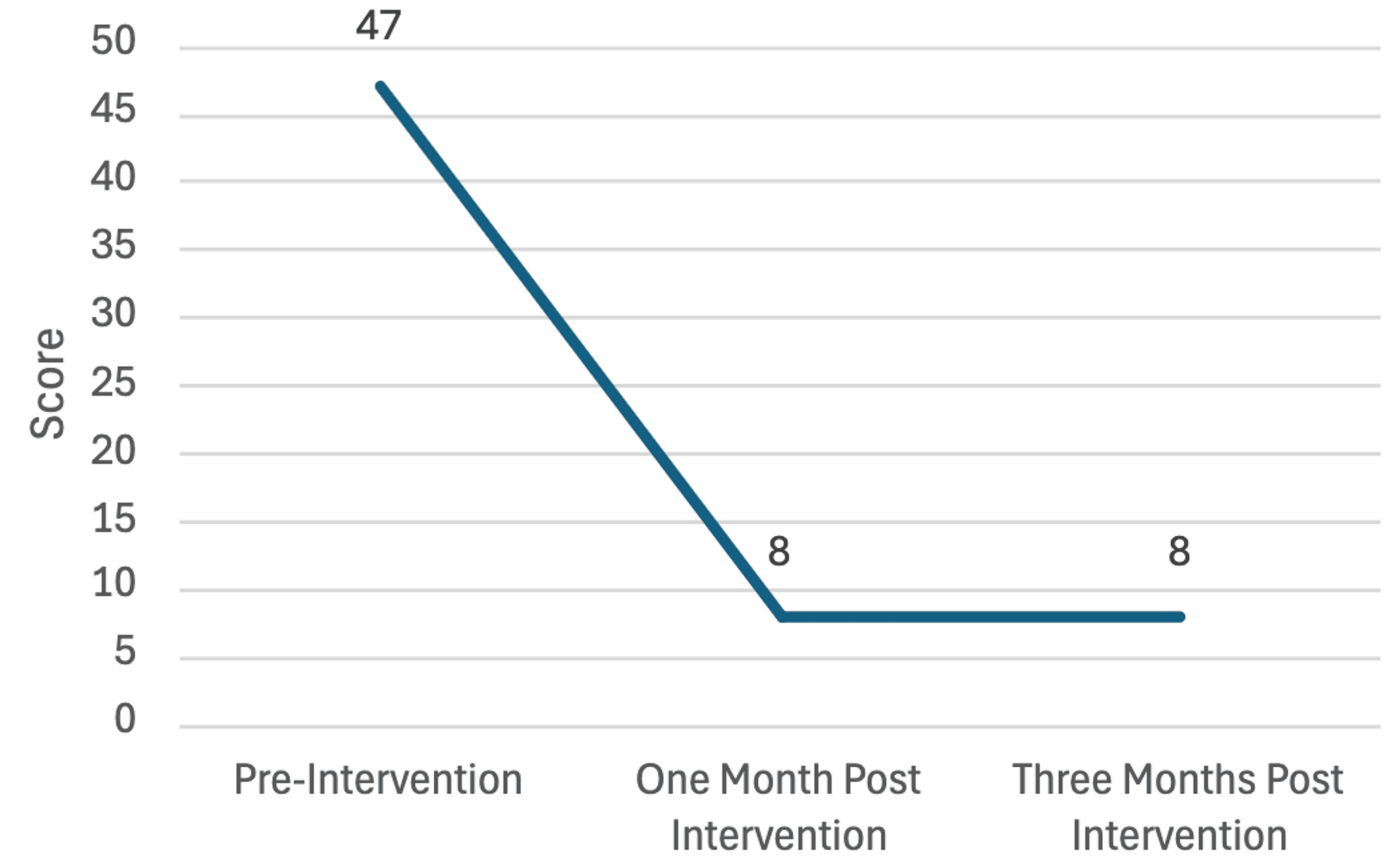
Total WOMAC scores prior to, one month, and three months after genicular artery embolization WOMAC: Western Ontario and McMaster Universities Osteoarthritis Index

**Figure 2:**
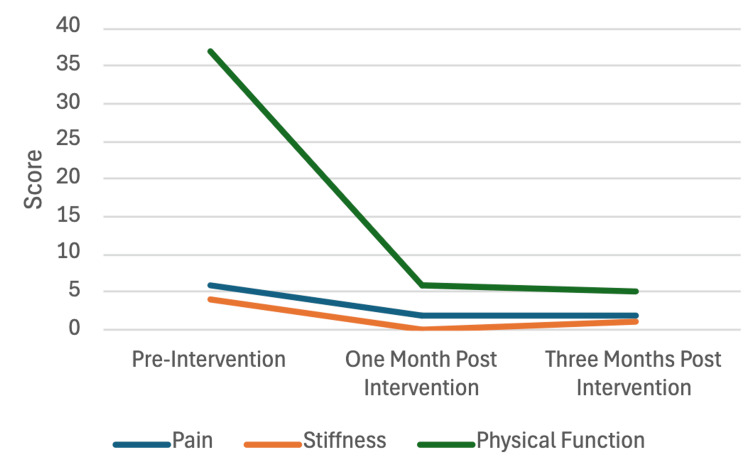
WOMAC component scores, including pain, stiffness, and physical function prior to, one, and three months after genicular artery embolization WOMAC: Western Ontario and McMaster Universities Osteoarthritis Index

Physical exam revealed a well-appearing, age-appropriate, non-obese male with an antalgic gait. Evaluation of the right knee showed well-healed midline and lateral incisions from previous open reduction and internal fixation of a distal femur fracture and TKA. The patient had an active and passive right knee range of motion from 20 to 95 degrees. Motor and sensory components of the exam were unremarkable bilaterally. Right knee imaging demonstrated well-aligned and stable components with a retained lateral locking plate. The patient was referred to orthopedic trauma for evaluation for hardware removal for potential impingement of soft tissue, as well as interventional radiology for assessment for geniculate artery embolization. Consultation with orthopedic traumatology concluded that the hemarthrosis was secondary to hemophilia; therefore, no hardware removal was completed. The patient saw an interventional radiologist who recommended GAE. Prior to GAE, the WOMAC score was 47 (pain 6/20; stiffness 4/8; physical function 37/68) (Figures 1-2, Table 1) [8].

**Table 1:** WOMAC raw scores pre-procedure, one month post-procedure, and three months post-procedure

	WOMAC Total	Pain	Stiffness	Physical Function
Pre Operative	47	6	4	37
One Month Post	8	2	0	6
Three Months Post	8	2	1	5

**Figure 3:**
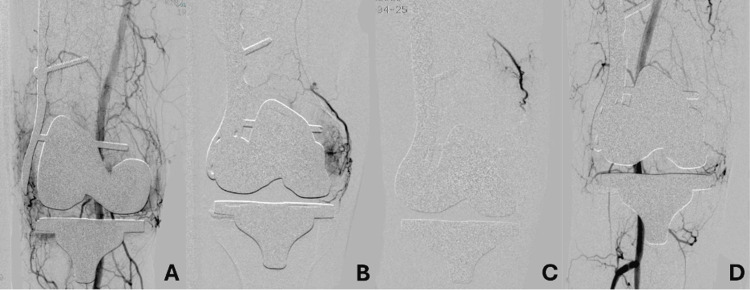
Evaluation of genicular arteries before and after selective embolization (A) Pre-interventional angiography-guided assessment of genicular arteries in a 62-year-old male with hemophilia A and a history of total knee arthroplasty demonstrating hyperemia of the right knee. (B) Intra-embolization of the descending geniculate artery via angiography. (C) Post-intervention angiography of the descending geniculate artery branch demonstrating appropriate filling defect. (D) Final post-intervention assessment of genicular artery embolization success in a patient with total knee arthroplasty.

GAE was performed by a board-certified interventional radiologist in the office setting. Thirty minutes prior to the procedure, the patient received eloctate 40 units/kg +/- 10% (3000u). Right groin access was obtained in an antegrade direction, and multistation right lower extremity angiography was performed through a 5F sheath. Selective catheterization of the right superficial femoral artery and geniculate artery ostia was performed with a 4F JR3.5 catheter (Merit Medical Systems, Inc., South Jordan, US) followed by distal catheterization with a 016 Fathom wire (Boston Scientific, Marlborough, US) and 2.0F Progreat Alpha (Terumo Global, Tokyo, Japan) microcatheter. In total, the right descending geniculate, superior medial geniculate, superior lateral geniculate, and anterior tibial recurrent geniculate arteries were selectively catheterized and then embolized to near stasis with 100-300 uM embospheres (Merit Medical) (Figures 3A-3D). Afterward, confirmation of no arteriovenous shunt or significant non-target location (large cutaneous branch, popliteal collateral) was evaluated. Twenty-four hours after the procedure, the patient received another dose of eloctate at 40 units/kg +/- 10% (3000u).

The patient was seen the day after the procedure for an access site groin check without complication. No limitations were given regarding the patient's activity level or range of motion of the affected knee. The WOMAC score at one month post-GAE was 8 (pain 2/20; stiffness 0/8; physical function 6/68). The final WOMAC score at three months post GAE was 8 (pain 2/20; stiffness 1/8; physical function 5/68) (Figures 1-2, Table 1). The patient did not undergo hardware removal due to symptom resolution following GAE. At the final follow-up of three months, the patient had no complaints of knee pain or stiffness. Additionally, there was no recurrence of effusion during the follow-up period as determined by ultrasound and clinical exam. Final active and passive range of motion was 15 to 95 degrees.

## Discussion

The prevalence of chronic pain following TKA in the general population is estimated to be 20% [9]. Pain that begins or increases in intensity following tissue trauma during surgery, such as TKA, which persists beyond three months after the index procedure, is classified as chronic postsurgical pain (CPSP) [10]. In the setting of this case report, our patient experienced CPSP following TKA due to recurrent hemarthrosis in the setting of hemophilia A. Patients with hemophilia are roughly 6% more susceptible to recurrent hemarthrosis than those without hemophilia following TKA [3]. RE in patients after TKA can cause significant pain and limitation to function while increasing risk for infection, which can be avoided by early diagnosis and intervention [4-6,11].

After a thorough workup to rule out infection, our patient was offered and elected to undergo genicular artery embolization to treat RE. The patient experienced a 83% decrease in WOMAC score at the 1-month mark. The decreased WOMAC score was maintained through the three-month follow-up period. 

In prior studies, GAE has continued to show reliable outcomes for the treatment of RE. In a review conducted by Kolber et al., GAE reported technical success of 99% and only 11% recurrence at variable timepoints. A case series conducted by Ogilvie et al. reported successful outcomes with GAE in five patients experiencing RE, emphasizing its role as a minimally invasive procedure with favorable clinical outcomes [12,13]. In an updated meta-analysis by Koullias et. al. in 2023, 212 patients underwent GAE for RE, demonstrating a GAE success rate of 95.2%, with symptomatic relief in 72.6% of patients. Over an average of 48 months, only 22% of cases reported recurrent effusion; however, there was no mention of the coagulation status of patients [14]. Chau et al. conducted a follow-up, prospective, single-center study assessing the utility of GAE for patients with CPSP. The authors collected Knee Injury and Osteoarthritis Outcome Score (KOOS) pain scores and VAS scores at the pre-treatment, three-month, and six-month follow-up. On average, about two abnormal, hyperemic arteries were embolized. Though there was a transient increase in pain within 2 weeks post-procedure, the authors found that at the 6-month follow-up, 55% patients reached a minimal clinically meaningful change in pain and 73% in quality of life [15]. Additionally, Tat-Sing Law and McClure presented three cases where therapeutic embolization effectively resolved recurrent hemarthrosis after knee arthroplasty at a median follow-up of two years [16]. Their findings align with those of Luyckx et al. and Lutz et al., who conducted retrospective studies demonstrating the safety and efficacy of GAE in recurrent hemarthrosis cases. Notably, Luyckx et al. showed that repeated GAE procedures can be safely performed without significant complications [7,17]. Furthermore, Heller et al. discussed the dual role of GAE in treating recurrent hemarthrosis, reaching clinical success of roughly 90% while commenting on several studies utilizing GAE to manage osteoarthritis-related knee pain, providing additional therapeutic potential for this intervention [18].

While GAE has shown promising results, not all cases result in complete resolution and are not without complications. Guevara et al. highlighted that technically successful embolization does not always equate to clinical success, suggesting that other factors, such as coagulopathy or synovial hypertrophy, may contribute to persistent symptoms [19]. Though our case demonstrates the utility of GAE in a PWH, comprehensive patient evaluation and individualized treatment planning is important. Periprosthetic joint infection (PJI) is a devastating complication following TKA. Kolber et al. reported an incidence of PJI at 2%. The authors also reported on minor complications, including transient cutaneous ischemia, post-embolization pain, and skin ulceration, all of which resolved within 4 weeks of GAE [12]. To decrease complications, proper perioperative management of thrombotic and bleeding risks is essential. Patel et al. provided consensus guidelines for the periprocedural management of such risks in patients undergoing image-guided interventions [20]. Their guidelines, endorsed by major interventional radiology societies, highlight the importance of balancing anticoagulation therapy with bleeding risk in post-arthroplasty patients.

The current study utilizes WOMAC scores, which limits direct comparisons to the former studies. However, we have similarly noticed improvements in patient-reported outcomes. Lutz et al. report similar improvements in WOMAC scores. Additionally, Heller et al. report on the efficacy of GAE in patients with osteoarthritis, assessing improvement with the WOMAC score, further demonstrating the validity and reliability of this scoring method [7,17].

Recurrent effusion following TKA requires a multidisciplinary approach. While GAE continues to prove its safety and efficacy in the treatment of RE after TKA, a multi-center randomized controlled trial is required to provide sufficient support for efficacy, specifically in patients with coagulopathies. Much of the literature published on GAE has no mention of efficacy in patients with coagulopathies. This report is unique in that it describes the complete resolution of RE in PWH after TKA.

## Conclusions

Hemarthrosis following TKA is a common and significant complication for PWH. In this case report, GAE provided an impressive decrease in the total WOMAC score in the post-procedure period and completely resolved recurrent effusion, as demonstrated by ultrasound. Therefore, we propose a safe, novel, and accessible treatment for hemarthrosis after TKA in PWH. To our knowledge, there is no established standard of care for this problem within this population. Our study demonstrates improved function and satisfaction in PWH who have RE leading to CPSP following TKA.
